# Research hotspots and emerging trends of mesenchymal stem cells in cardiovascular diseases: a bibliometric-based visual analysis

**DOI:** 10.3389/fcvm.2024.1394453

**Published:** 2024-05-30

**Authors:** Zhihang Jiang, Jiajing Yu, Houle Zhou, Jiaming Feng, Zehui Xu, Melisandre Wan, Weiwei Zhang, Yuqing He, Chengyao Jia, Shuijin Shao, Haidong Guo, Baonian Liu

**Affiliations:** ^1^ Department of Anatomy, School of Chinese Integrative Medicine, Shanghai University of Traditional Chinese Medicine, Shanghai, China; ^2^ Department of Preventive Medicine, College of Public Health, Shanghai University of Traditional Chinese Medicine, Shanghai, China; ^3^ Guanghua School of Stomatology, Sun Yat-Sen University, Guangzhou, China

**Keywords:** mesenchymal stem cells, cardiovascular diseases, exosome, therapeutic target, bibliometric study

## Abstract

**Background:**

Mesenchymal stem cells (MSCs) have important research value and broad application prospects in cardiovascular diseases (CVDs). However, few bibliometric analyses on MSCs in cardiovascular diseases are available. This study aims to provide a thorough review of the cooperation and influence of countries, institutions, authors, and journals in the field of MSCs in cardiovascular diseases, with the provision of discoveries in the latest progress, evolution paths, frontier research hotspots, and future research trends in the regarding field.

**Methods:**

The articles related to MSCs in cardiovascular diseases were retrieved from the Web of Science. The bibliometric study was performed by CiteSpace and VOSviewer, and the knowledge map was generated based on data obtained from retrieved articles.

**Results:**

In our study, a total of 4,852 publications launched before August 31, 2023 were accessed through the Web of Science Core Collection (WoSCC) database via our searching strategy. Significant fluctuations in global publications were observed in the field of MSCs in CVDs. China emerged as the nation with the largest number of publications, yet a shortage of high-quality articles was noted. The interplay among countries, institutions, journals and authors is visually represented in the enclosed figures. Importantly, current research trends and hotspots are elucidated. Cluster analysis on references has highlighted the considerable interest in exosomes, extracellular vesicles, and microvesicles. Besides, keywords analysis revealed a strong emphasis on myocardial infarction, therapy, and transplantation. Treatment methods-related keywords were prominent, while keywords associated with extracellular vesicles gathered significant attention from the long-term perspective.

**Conclusion:**

MSCs in CVDs have become a topic of active research interest, showcasing its latent value and potential. By summarizing the latest progress, identifying the research hotspots, and discussing the future trends in the advancement of MSCs in CVDs, we aim to offer valuable insights for considering research prospects.

## 1 Introduction

Cardiovascular disease (CVD) is a pressing public health concern that remains the leading cause of death globally (1). However, traditional treatment methods for cardiovascular diseases, such as various drug therapies and surgical interventions, may result in significant adverse reactions or disease recurrence (2). Recent studies have highlighted the promising role of mesenchymal stem cells (MSCs) in the treatment of several kinds of CVDs (3–6).

MSCs, a type of cell with self-renewal ability, multi-differentiation potential and immune regulation functions, exist in a variety of tissues (7–9). MSCs stain positive for a series of cell surface markers including CD73, CD44, CD90, and CD105, yet stain negative for the hematopoietic markers such as CD34 and CD45 and HLA-DR (10). MSCs can differentiate into various cell types including chondrocytes, adipocytes, cardiomyocytes, muscle cells, etc., which makes them valuable for both in-vivo and in-vitro applications (10–14). MSCs can exhibit immunomodulatory function through cell-to-cell contact, and perform the release of cytokines to inhibit the proliferation and immune response of *T* cells (15). Based on the characteristics that have both regenerative and immune regulatory abilities, MSCs can be widely applied in clinical research and can show extremely high research value. The differentiation ability of MSCs can be utilized for stem cell therapy of cardiovascular diseases and it can to some extent avoid the occurrence of adverse reactions (16). Shin et al. discovered that the MSCs intravenously injected can specifically migrate to the inflammatory site caused by ischemic injury, and it has been confirmed that administration of MSCs after myocardial infarction injury can enhance adenosine production through CD73 activity and reduce reactive oxygen species (ROS) formation following MI/R (17). Under appropriate conditions, MSCs can differentiate into functional cardiomyocytes (18–20). For example, 5-azacytidine can induce MSCs to express proteins commonly associated with cardiomyocytes, including beta-MHC, desmin, and alpha-cardiac actin, thereby showing potential to repair injured myocardium (21). MSCs can also promote angiogenesis through differentiation. Quevedo et al. performed catheter-based transendocardial injections of MSCs on swine and found that MSCs can differentiate into vascular smooth muscle and endothelial cells, contributing to the formation of vessels of all sizes (22).

Bibliometric analysis evaluates and quantifies literature information by analyzing different features of articles, aiming to identify global research trends (23). It typically uses scientific articles found in the databases as input to create interactive visual representations for statistical analysis (24). The primary research issues and frontiers can be identified by swiftly assembling a large number of published articles, sketching and visualizing the structure and dynamics of a research field, and then conducting further analysis (25). Bibliometrics has unparalleled advantages in analyzing research hotspots and frontiers compared to other methods due to its specialization in identifying core researchers, institutions, and countries in the field, and their collaborative relationships as well (26). Using software for bibliometric analysis, a knowledge map is created by thoroughly reviewing the literature on the suggested research dimensions (27). Due to the advantages of this method, more and more scholars are using bibliometric analysis in various fields of medicine, such as gynecology (28), hypertension (29), lung diseases (30), inflammatory bowel disease (31), aortic disease (32), and autoimmune diseases (33).

This research aims to analyze the trends and hotspots in the utilization of MSCs for CVDs over the past decade using tools like CiteSpace and VOSviewer. By creating a knowledge map based on bibliometric analysis, the study provides insights into the latest progress, evolution, and future directions of MSC research in CVDs, benefiting both basic research and clinical treatment.

## 2 Methods

### 2.1 Data sources and searches

**Figure 1:**
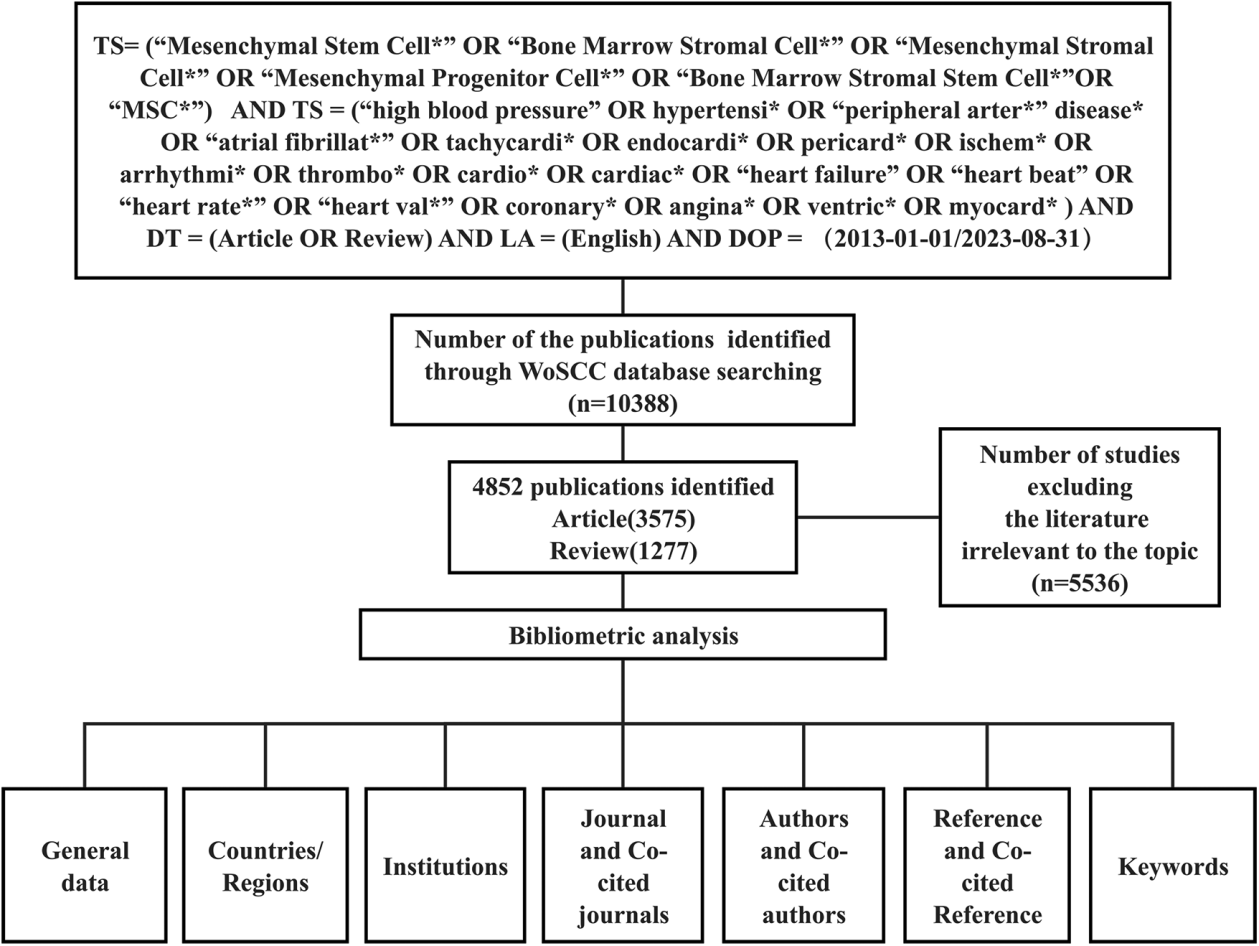
Flowchart of literature selection.

The Science Citation Index Expanded (SCI-E) and Social Science Citation Index (SSCI) of the core database of the document information index database Web of Science were selected as the target databases for bibliometric analysis. The search formula was set to TS = (“Mesenchymal Stem Cell*” OR “Bone Marrow Stromal Cell*” OR “Mesenchymal Stromal Cell*” OR “Mesenchymal Progenitor Cell*” OR “Bone Marrow Stromal Stem Cell*”OR “MSC*”) AND TS = (“high blood pressure” or hypertensi* or “peripheral arter*” disease* or “atrial fibrillat*” or tachycardi* or endocardi* or pericard* or ischem* or arrhythmi* or thrombo* or cardio* or cardiac* or “heart failure” or “heart beat” or “heart rate*” or “heart val*” or coronary* or angina* or ventric* or myocard*) AND DT = (Article OR Review) AND LA = (English) AND DOP = (2013-01-01/2023-08-31). A total of 10,388 publications were originally identified through WoSCC database searching. Only articles and reviews published in English that met the theme of MSCs in CVDs were selected for further analysis, while book chapters, proceedings papers, meeting abstracts, corrections, editorials, duplicates, retractions, or unpublished articles were excluded. Two independent investigators (Jiajing Yu and Houle Zhou) retrieved and filtered the publications to ensure the accuracy of the scientometric analysis. After removing literature that does not match this topic (*n* = 5,536), 4,852 studies were identified as eligible for our study, consisting of articles (*n* = 3,575) and reviews (*n* = 1,277). The detailed filtering process is demonstrated in Figure 1.

### 2.2 Data analysis

The software used in the study includes VOSviewer [version 1.6.20(0), Leiden University, Leiden, Netherlands], CiteSpace (version 6.2. R5, Chaomei Chen, Drexel University, Philadelphia, PA, United States), Microsoft Excel for Mac [version 16.78.3(23102801), WA, United States], the R language based Bibliometrix Package (4.1.3 Package) and Scimago Graphica (Beta 1.0.36). These applications were used to analyze the 10,388 exported articles and data information including the number and year of publications, total and average citations, titles, countries and institutions, authors, journals, keywords, and references. VOSviewer was used to construct and view bibliometric maps, which has functionality for zooming, scrolling, and searching (34). The VOSviewer map depicts weighting by nodes, collaborative relationships by links, and link strength by link thickness. To distinguish clusters, different colors are used in the module visualizing network. The Bibliometric package (4.1.3 package) based on R language is used to analyze three-field plots, displaying the relationships between co-cited references, authors, and keywords.

## 3 Results

### 3.1 Annual publication outputs

**Figure 2:**
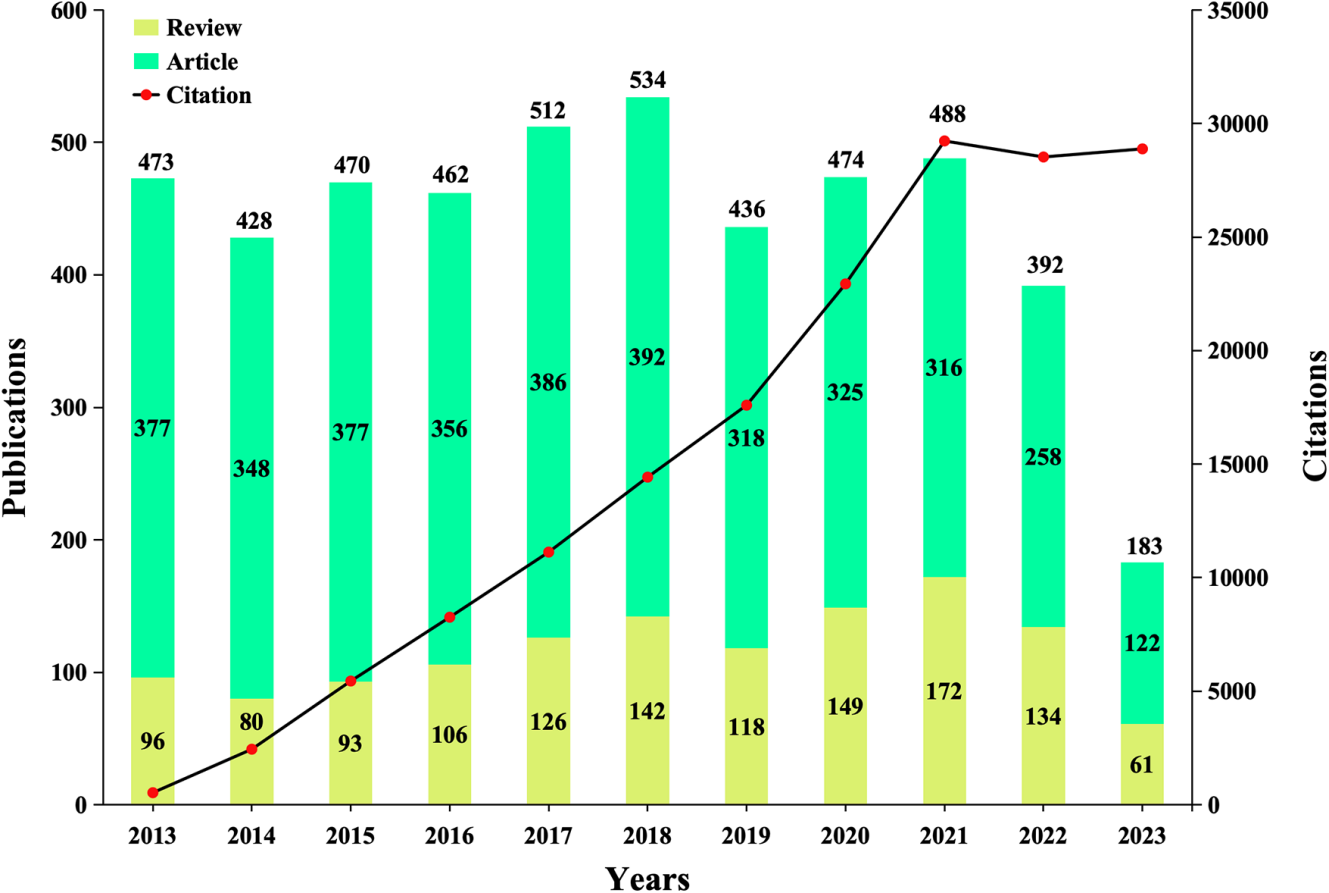
Trends in the growth of publications (reviews and articles), and citations worldwide from 2013 to 2023. Due to the need for article revisions, the citation data of the articles filtered was updated, and the latest citation data displayed is as of April 23, 2024.

Figure 2 indicates the change in annual publications (reviews and articles) and citation frequency, which witnessed the progress of this field. From 2013 to 2023, the annual publications on MSCs in cardiovascular diseases included 4,852 related articles, generally presenting an ascending trend. The number of papers published on MSCs in CVDs experienced an upturn from 2013 to 2018, then declined from 2019 to 2022 compared to the previous years. The articles we obtained for this study were published before August 31, 2023. We speculate that by the end of 2023, the total number of publications will be equal to or less than the number in 2022. In addition, Figure 2 depicts the upward trend of citation frequency from 2013 to 2021. We have updated the citation data and the latest citation data displayed is as of April 23, 2024. The citations in 2022 had a modest drop compared to 2021. However, the citation count of 183 papers in the first 8 months of 2023 has exceeded the citation count of 392 articles in the entire year of 2022. This indicates that the interest and influence of this study in this field will continue to grow.

### 3.2 Contribution of countries/regions

**Figure 3:**
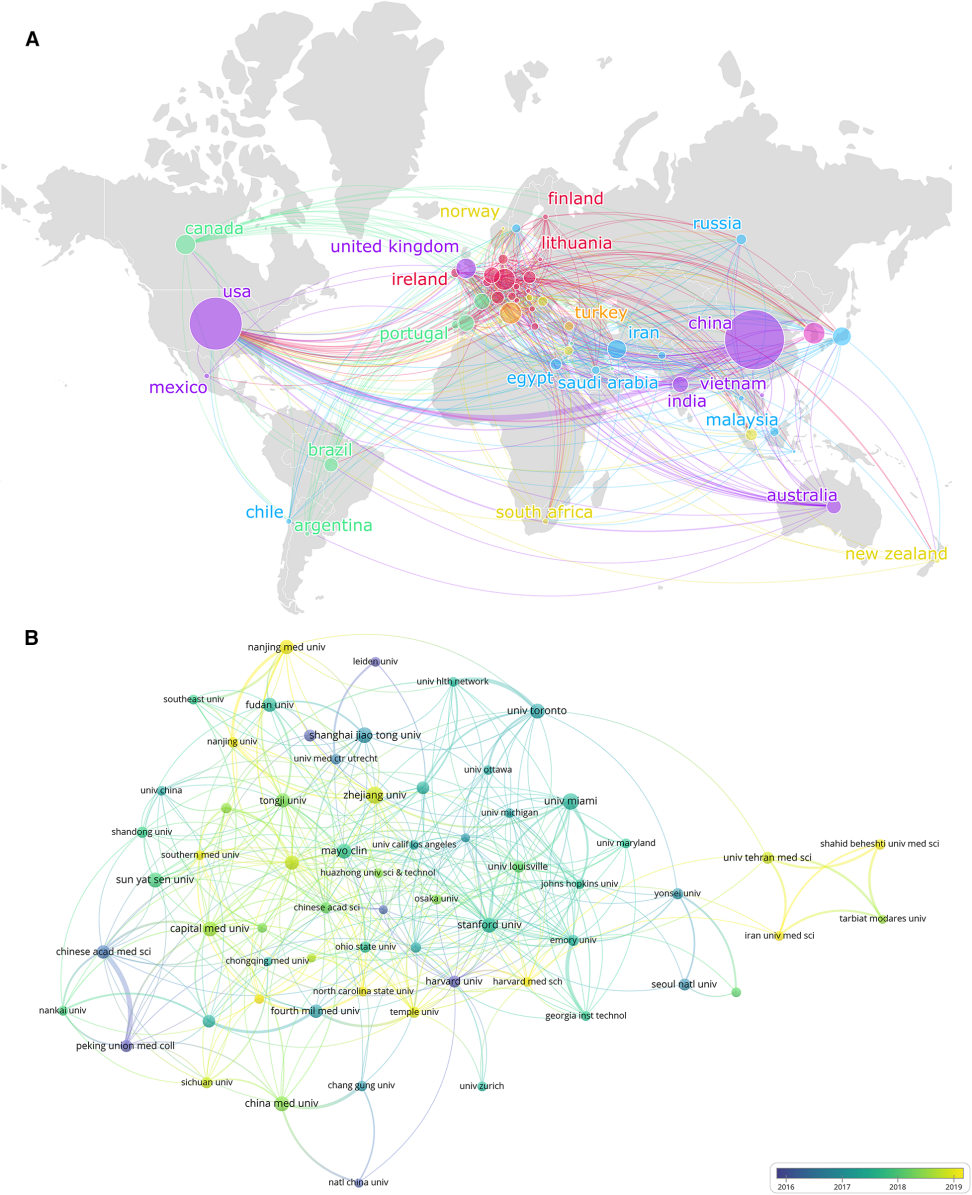
The collaboration of countries/regions and institutions in the field of MSCs in CVDs. (**A**) Collaboration map of the publications and co-occurrence network of countries/regions. (**B**) Institutional cooperation/contributions to publications.

Table 1 ranked 10 high-productivity countries that contributed to publications of MSCs in CVDs. China and the United States stand out as the two primary contributors in this research field, showcasing a contrast in the number of publications and citations compared to other countries. China leads with the highest number of publications (1,735 documents), which represents 37.36% of the total. The United States follows closely behind with 1,350 publications, accounting for 29.07% of the research output. Additionally, Italy, Germany, South Korea, and the United Kingdom have also played substantial roles in this field, with publication numbers of 233 (5.02%), 226 (4.87%), 226 (4.87%), and 201 (4.33%) respectively. While Canada, Iran, Japan, and the Netherlands have made noteworthy contributions, their publication numbers remain below 200. As is shown in Figure 3A, nodes of different sizes represent countries with different levels of participation, and different colors represent clusters of close cooperation. For example, the United States and China have collaborated closely in this research field. Interestingly, the USA has garnered the highest number of citations, totaling 62,761, while China closely follows with 51,896 citations, slightly below the USA. This data suggests that USA (1,350 documents, 62,761 citations, and 825 total link strength) stands out as the country with the most extensively collaborative network in publications related to MSCs in CVDs.

**Table 1:** Top 10 countries and institutions that contributed to publications of MSC in CVD.

Top 10 countries that contributed to publications of MSC in CVD						
Rank	Country	Document	Percentage (%)	Citation	Citation/document	Total link strength
1	China	1,735	37.36	51,896	29.91	440
2	United States	1,350	29.07	62,761	46.49	825
3	Italy	233	5.02	8,591	36.87	230
4	Germany	226	4.87	10,462	46.29	252
5	South Korea	226	4.87	7,903	34.97	99
6	United Kingdom	201	4.33	8,443	42.00	211
7	Canada	196	4.22	7,253	37.01	122
8	Iran	175	3.77	4,127	23.58	98
9	Japan	171	3.68	5,384	31.49	99
10	Netherlands	131	2.82	6,810	51.98	172
Top 10 institutions that contributed to publications of MSC in CVD						
Rank	Institution (Country)	Document	Percentage (%)	Citation	Citation/document	Total link strength
1	Zhejiang University (China)	90	12.47	3,103	34.48	32
2	University of Miami (United States)	80	11.08	5,443	68.04	45
3	Shanghai Jiao Tong University (China)	76	10.53	2,803	36.88	27
4	Sun Yat-sen University (China)	72	9.97	2,066	28.69	22
5	Stanford University (United States)	70	9.70	2,558	36.54	66
6	University of Toronto (Canada)	69	9.56	2,132	30.90	55
7	Capital Medical University (China)	68	9.42	1,539	22.63	52
8	China Medical University (China)	67	9.28	1,277	19.06	40
9	Mayo Clinic (United States)	67	9.28	2,831	42.25	23
10	Nanjing Medical University (China)	63	8.73	1,561	24.78	39

### 3.3 Contribution of institutions

Table 1 also displayed the top 10 most productive institutions in the field. Furthermore, the VOSviewer was applied to cluster the cooperating organizations, and formed a clustering diagram (Figure 3B). Zhejiang University leads in publication volume with 90 articles, establishing itself as a key contributor in terms of the number of publications. Following closely come the University of Miami (80 articles), Shanghai Jiao Tong University (76 articles), and Sun Yat-sen University (72 articles).

Notably, the University of Miami stands out with the highest number of citations, indicating a relatively high quality of articles produced by the institution. In Figure 3B, institutions closer to the yellow color spectrum are depicted to be more active recently, while those closer to the blue spectrum indicate a higher level of activity in the past.

### 3.4 Journal and co-cited journals

The top 10 most productive journals and co-cited journals in the field of MSCs in CVDs are ranked in Table 2. The top three journals in number of publications were Stem Cell Research & Therapy (184 articles), International Journal of Molecular Sciences (131 articles) and Stem Cells International (128 articles), each of which possessed more than 120 documents. While referring to the H-index of the top 10 productive journals, Circulation Research (48 H-index) ranked first, which reflects the high quality of its production and academic influence, followed by Stem Cell Research & Therapy (47 H-index), which were the only two journals with an H-index exceeding 40. The influence factor is one of the indicators to measure the influence of a journal, which reflects the frequency of article citations. According to Table 2, the journal with the highest impact factor is Circulation Research, reaching 20.1. This indicates that the articles in this journal have great influence and importance in the academic community. Regarding the total link strength, Stem Cell Research & Therapy (13,676 citations and 1,312,401 total link strength) and International Journal of Molecular Sciences (13,430 citations and 1,308,475 total link strength) featured the most co-citations along with others, with a total link strength that surpassed 1,300,000. By analyzing these data, we can figure out which journals have more influence and importance in the academic community, as well as the correlation between them. For example, Circulation and Circulation Research are two widely cited journals, which also show a high link strength. This indicates that they have a great influence in the field of MSCs in CVDs, and there may be a cooperative relationship or mutual reference.

**Table 2:** Top 10 journals and co-cited journals by papers of MSC in CVD.

Rank	Journal	Document	Total citation	Citation/document	Total link strength	IF 2022	H-index	Rank	Co-cited journal	Citations	Total link strength
1	Stem cell Research & Therapy	184	7,284	39.59	2,207	7.5	47	1	Circulation	13,676	1,312,401
2	International Journal of Molecular Sciences	131	2,624	20.03	1,088	5.6	26	2	Circulation Research	13,430	1,308,475
3	Stem Cells International	128	4,869	38.04	1,416	4.3	36	3	Biomaterials	8,308	930,109
4	PLoS ONE	102	3,182	31.20	662	3.7	30	4	Stem Cells	7,041	678,591
5	Scientific Reports	89	2,777	31.20	569	4.6	31	5	PLoS ONE	6,901	778,186
6	Stem Cells Translational Medicine	76	3,105	40.86	798	6.0	33	6	Proceedings of the National Academy of Sciences of the United States of America	5,804	687,141
7	Biomaterials	72	3,733	51.85	616	14.0	36	7	Journal of the American College of Cardiology	5,294	529,744
8	Circulation Research	68	6,333	93.13	1,825	20.1	48	8	Nature	5,021	602,297
9	Stem Cells	64	4,437	69.33	680	5.2	34	9	Stem Cell Research & Therapy	4,323	464,528
10	Cell Transplantation	57	1,892	33.19	410	3.3	22	10	Cardiovascular Research	4,005	437,145
10	Frontiers in Cardiovascular Medicine	57	982	17.23	592	3.6	17				

**Figure 4:**
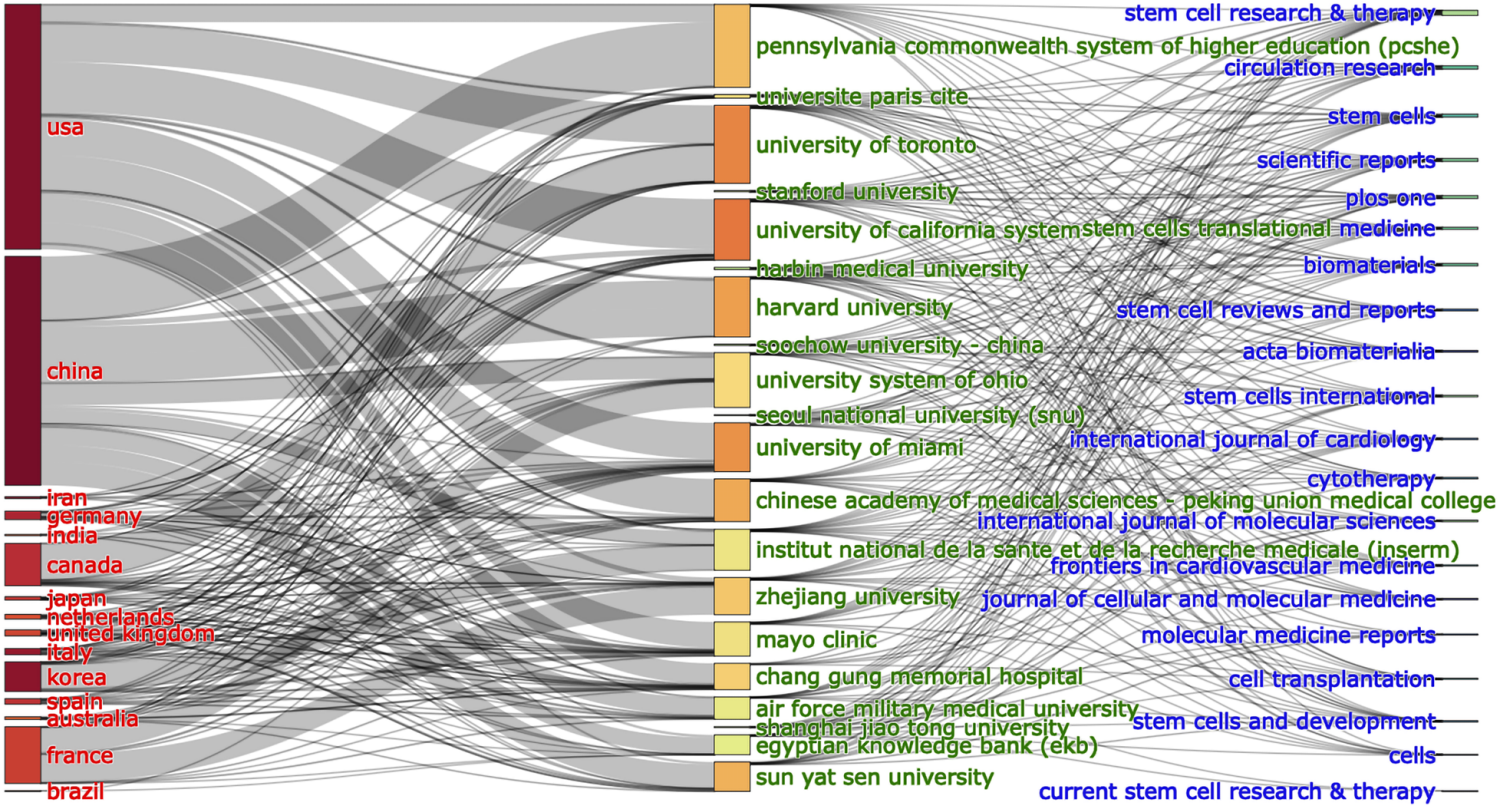
The relationship of the countries, institutions, and research journals that produced articles in an alluvial flow map based on MSCs in CVDs.

The bond between countries, institutions and journals based on a three-field plot for MSCs in CVDs is demonstrated in Figure 4. China exerts the greatest influence among the 20 countries, closely followed by the USA. The six dominant institutions lying foremost on the list are Chang Gung Memorial Hospital, the University of California System, Harvard University, Pennsylvania Commonwealth System of Higher Education (PCSHE), the University of Toronto and the University of Miami. Notably, from the last two columns of the chart, we can learn the information of the collaborations between institutions and journals. The six journals with the most connections are Stem Cell Research & Therapy, Circulation Research, Stem Cells, Scientific Reports, Plos One, and Stem Cells Translational Medicine (Figure 4).

### 3.5 Authors and co-cited authors

Table 3 lists the top 10 authors who contributed most to the literature on MSCs in CVDs. Hare Joshua M. (49 Documents, 3,488 citations, and 33 H-index) ranks first with the most citations per document among the top ten authors in this field, reaching 71.18, indicating that the articles he published have been highly influential. Bolli Roberto, Zhu Wei, and Cheng Ke ranked second, third, and fourth, each of whom published 27 articles. Other top ten authors also published over 20 articles. These authors have made outstanding contributions to the field of MSCs in CVDs. Their articles have been widely cited and developed a high H-index, indicating that their research has been widely recognized by peers (Table 3).

**Table 3:** Top 10 authors and co-cited authors in the field of MSC in CVD.

Top 10 authors in the field of MSC in CVD					
Rank	Author	Document	Citations	Citation/document	H-index
1	Hare, Joshua M	49	3,488	71.18	33
2	Bolli, Roberto	27	1,707	63.22	17
2	Zhu, Wei	27	1,581	58.56	20
2	Cheng, Ke	27	1,560	57.78	20
5	Shen, Zhenya	26	1,464	56.31	16
5	Kastrup, Jens	26	925	35.58	19
7	Hu, Xinyang	25	1,119	44.76	19
8	Cao, Feng	24	1,484	61.83	20
9	Wang, Jian'an	23	1,063	46.22	19
9	Yu, Hong	23	1,029	44.74	18
9	Bayes-Genis, Antoni	23	624	27.13	16
Top 10 co-cited authors in the field of MSC in CVD					
Rank	Author	Citations		Total link strength	
1	Gnecchi, M	893		30,501	
2	Hare, JM	861		31,683	
3	Pittenger, MF	634		16,861	
4	Dominici, M	570		15,552	
5	Williams, AR	516		17,017	
6	Bolli, R	486		20,499	
7	Perin, EC	476		23,677	
8	Menasché, P	464		24,510	
9	Lai, RC	458		17,483	
10	Caplan, AI	457		14,373	

As Table 3 shows, Gnecchi M. (893 citations, 30,501 total link strength) was the most high-yielding co-cited author. Hare Joshua M. with the highest total link strength, reaching 31,683, ranks second. His research focuses on MSCs in cardiomyogenesis and angiogenesis in postinfarct myocardium (35), reducing levels of apoptosis (36), increasing tissue perfusion (37), and stimulating the proliferation and differentiation of cardiac stem cells (38).

### 3.6 References and co-cited references

The top 10 references based on the number of citations are listed in Table 4, including three articles and seven reviews. The most cited reference written by Phinney Donald. et al. in 2017 was a review published in Stem Cells, entitled “Concise Review: MSC-Derived Exosomes for Cell-Free Therapy” (39), followed by a review entitled “Silk Fibroin Biomaterials for Tissue Regenerations” (40). Table 4 also illustrates the uppermost 10 co-cited references on the topic of MSCs in CVDs, with Dominici et al. (9), Pittenger et al. (41) and Hare et al. (42) ranking the highest.

**Table 4:** The top 10 references and co-cited references based on the number of citations.

Top 10 references based on the number of citations							
Rank	Citations	Title	First author	Document type	Year	Journal	IF (2022)
1	988	Concise Review: MSC-Derived Exosomes for Cell-Free Therapy	Phinney Donald G.	Review	2017	Stem Cells	5.2
2	888	Silk fibroin biomaterials for tissue regenerations	Kundu Banani	Review	2013	Advanced Drug Delivery Reviews	16.1
3	804	Mesenchymal stem cell-derived exosomes increase ATP levels, decrease oxidative stress and activate PI3 K/Akt pathway to enhance myocardial viability and prevent adverse remodeling after myocardial ischemia/reperfusion injury	Arslan Fatih	Article	2013	Stem Cell Research	1.2
4	717	Mesenchymal Stem Cell-derived Extracellular Vesicles: Toward Cell-free Therapeutic Applications	Rani Sweta	Review	2015	Molecular Therapy	12.4
5	693	Mechanobiology of YAP and TAZ in physiology and disease	Panciera Tito	Review	2017	Nature Reviews Molecular Cell Biology	112.7
6	643	Mesenchymal stem cells: a new trend for cell therapy	Wei Xin	Review	2013	Acta Pharmacologica Sinica	8.2
7	629	Using exosomes, naturally-equipped nanocarriers, for drug delivery	Batrakova Elena V.	Article	2015	Journal of Controlled Release	10.8
8	625	Perivascular Gli1+ Progenitors Are Key Contributors to Injury-Induced Organ Fibrosis	Kramann Rafael	Article	2015	Cell Stem Cell	23.9
9	592	Paracrine Mechanisms of Mesenchymal Stem Cell-Based Therapy: Current Status and Perspectives	Liang Xiaoting	Review	2014	Cell Transplantation	3.3
10	573	Mesenchymal Stem Cells for Regenerative Medicine	Han Yu	Review	2019	Cells	6.0
Top 10 co-cited references based on the number of citations							
Rank	Citations	Title	First author	Document type	Year	Journal	IF (2022)
1	570	Minimal criteria for defining multipotent mesenchymal stromal cells. The International Society for Cellular Therapy position statement	M. Dominici	Article	2006	Cytotherapy	4.5
2	413	Multilineage Potential of Adult Human Mesenchymal Stem Cells	Mark F. Pittenger	Article	1999	Science	56.9
3	373	Comparison of Allogeneic vs Autologous Bone Marrow–Derived Mesenchymal Stem Cells Delivered by Transendocardial Injection in Patients With Ischemic Cardiomyopathy	Joshua M. Hare	Article	2012	JAMA	120.7
4	328	Paracrine Mechanisms in Adult Stem Cell Signaling and Therapy	Massimiliano Gnecchi	Review	2008	Circulation Research	20.1
5	327	A Randomized, Double-Blind, Placebo-Controlled, Dose-Escalation Study of Intravenous Adult Human Mesenchymal Stem Cells (Prochymal) After Acute Myocardial Infarction	Joshua M. Hare	Article	2009	Journal of the American College of Cardiology	24.4
6	312	Human Mesenchymal Stem Cells Differentiate to a Cardiomyocyte Phenotype in the Adult Murine Heart	Catalin Toma	Article	2002	Circulation	37.8
7	275	Exosome secreted by MSC reduces myocardial ischemia/reperfusion injury	Ruenn Chai Lai	Article	2010	Stem Cell Research	1.2
8	264	Intracoronary cardiosphere-derived cells for heart regeneration after myocardial infarction (CADUCEUS): a prospective, randomised phase 1 trial	Raj R Makkar	Article	2012	The Lancet	168.9
9	254	Bone marrow cells regenerate infarcted myocardium	Donald Orlic	Article	2001	Nature	64.8
10	246	Adult Cardiac Stem Cells Are Multipotent and Support Myocardial Regeneration	Antonio P. Beltrami	Article	2003	Cell	64.5
10	246	Mesenchymal Stem Cells	Adam R. Williams	Review	2011	Circulation Research	20.1

**Figure 5:**
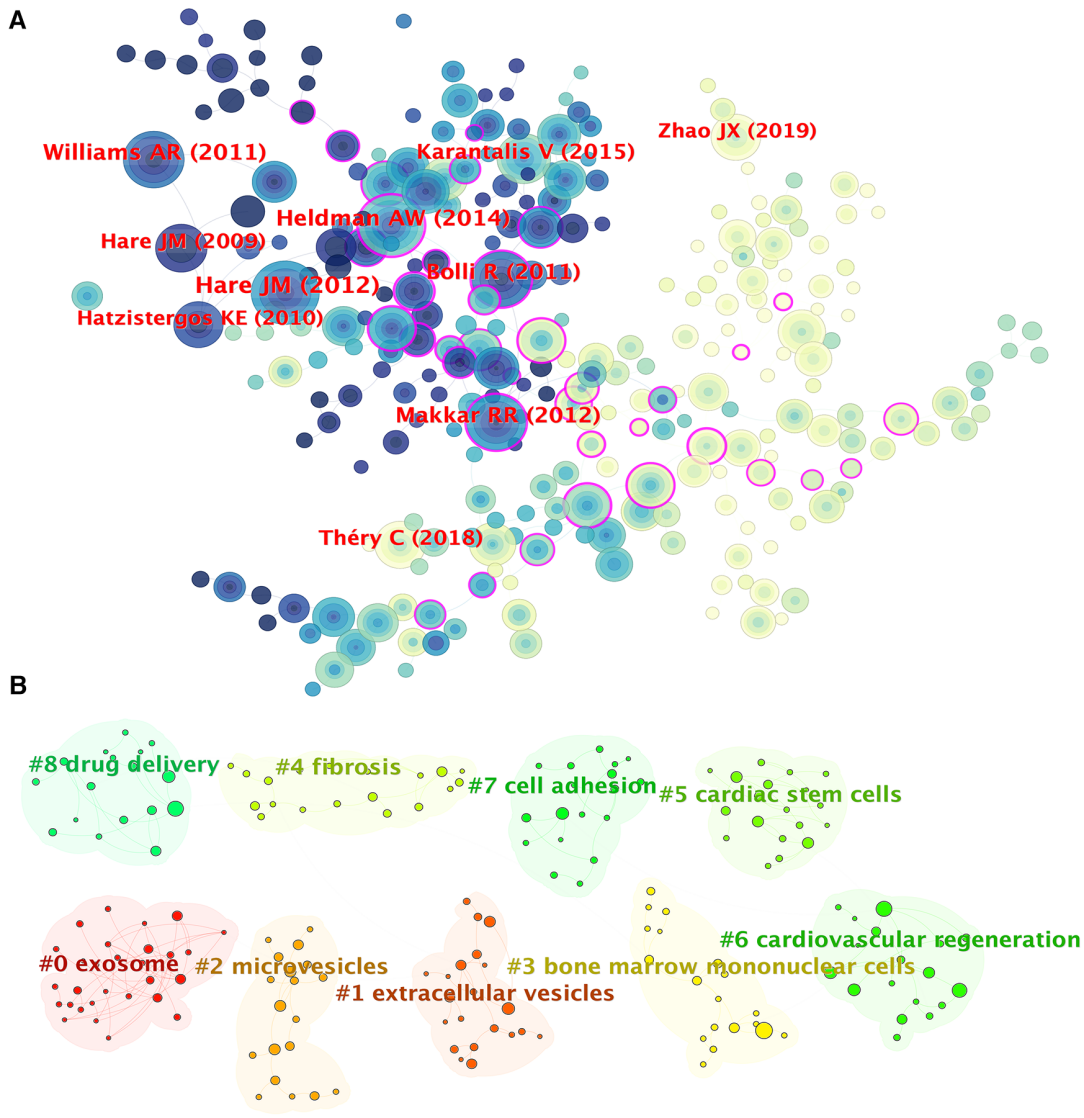
The collaboration of authors and co-cited references in the field of MSCs in CVDs. (**A**) The figure only shows the top 10 authors’ name. Cooperation network among the co-cited references in the studies of MSCs in CVDs. (**B**) A cluster of the co-cited references in the studies of MSCs in CVDs.

As was shown in Figure 5A, CiteSpace was used to extract the information of co-cited references. A cluster analysis was performed on the extracted information, as shown in Figure 5B. In Figure 5B, there are 18 clusters formed and 9 clusters showed in the figure: (0) exosomes, (1) extracellular vesicles (EVs), (2) microvesicles, (3) bone marrow mononuclear cells, (4) fibrosis, (5) cardiac stem cells, (6) cardiovascular regeneration, (7) cell adhesion, (8) drug delivery. The three clusters of exosomes, EVs and microvesicles have the highest burst strength. To some extent, these clusters reflect the main research focus in the fields of MSCs in CVDs.

**Figure 6:**
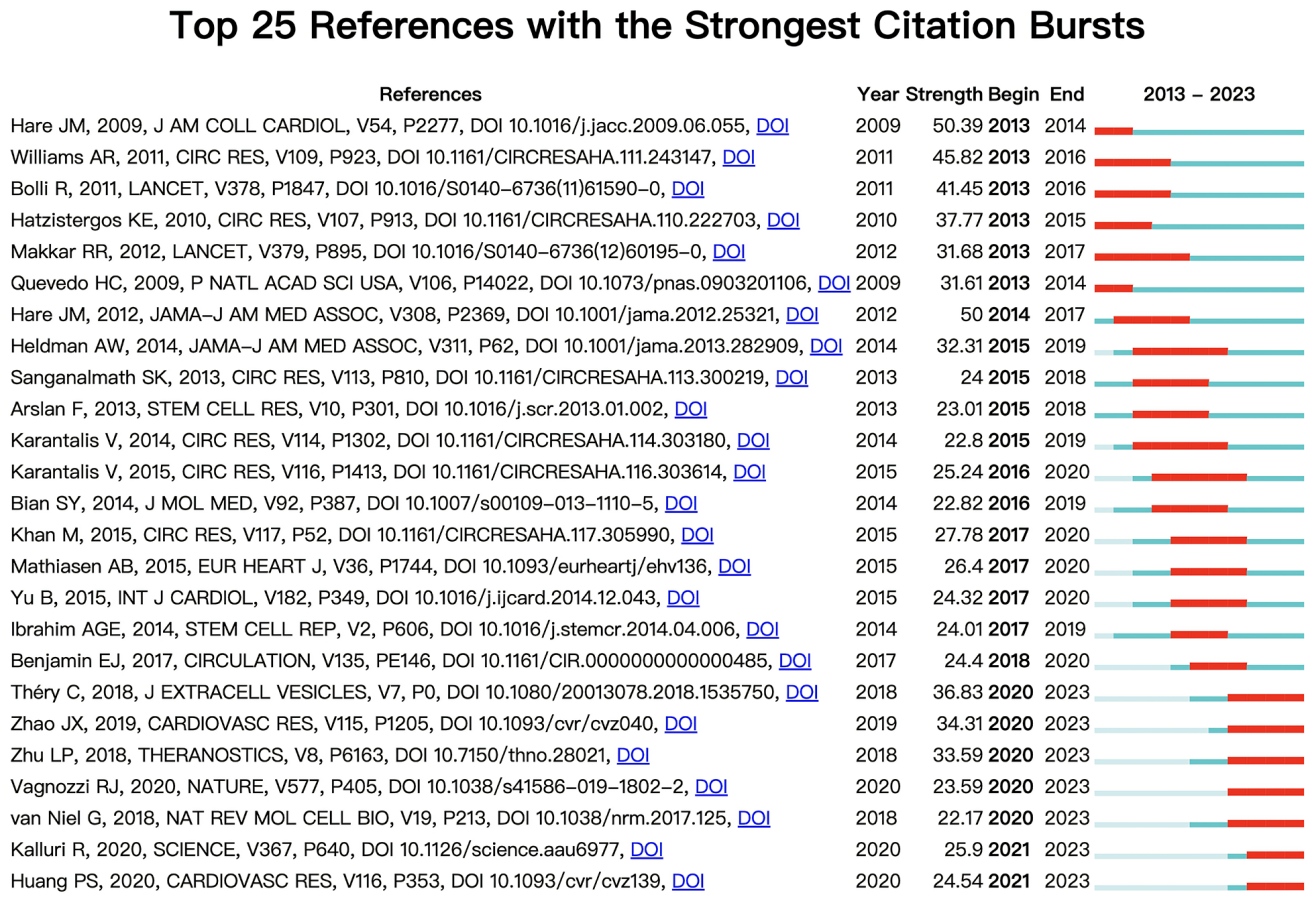
The top 25 references with the strongest citation bursts, based on citeSpace, that were involved in MSCs in CVDs between 2013 and 2023. The blue line represents the time from its first appearance to 2023, and the red line represents the burst time.

Figure 6 shows the top 25 references with the strongest citation bursts during 2013–2023. Through analysis of the references published over each specific period, we can identify the research hotspots during the corresponding time. As we can see in Figure 6, the first citation burst occurred between 2013 and 2014, and the latest reference with a strong citation burst was recorded from 2021 to the present. The highest burst strength was from Hare et al. (42) (50.39 strength), Williams et al. (2011) (45.82 strength), and Bolli et al. (2011) (41.45 strength). What's more, Kalluri et al. (2020) and Huang et al. (2020) have received wide attention in recent years (Figure 6).

### 3.7 Keyword analysis

The top 20 frequency keywords related to MSCs in CVDs are displayed in Table 5. “MSCs” (2,337 occurrences, 20,025 total link strength) was the keyword with the greatest number of occurrences. The second and third ranked keywords are “myocardial infarction” (793 occurrences, 7,317 total link strength) and “therapy” (722 occurrences, 7,035 total link strength). These data can help us understand which keywords are widely discussed and cited in the field of MSCs in CVDs, as well as their importance and research hotspots.

**Table 5:** Top 20 frequency keywords related to MSC in CVD.

Ranking	Keyword	Occurrences	Total link strength	Ranking	Keyword	Occurrences	Total link strength
1	Mesenchymal stem cells	2,337	20,025	11	Progenitor cells	563	5,456
2	Myocardial-infarction	793	7,317	12	Expression	562	4,891
3	Therapy	772	7,035	13	Heart	546	4,887
4	Transplantation	765	7,266	14	Exosomes	504	4,646
5	Stromal cells	760	7,091	15	Acute myocardial-infarction	402	3,950
6	Differentiation	720	6,300	16	Stem-cells	400	3,499
7	Bone-marrow	663	6,235	17	Repair	391	3,600
8	Angiogenesis	654	6,182	18	Extracellular vesicles	365	3,590
9	*in-vitro*	624	5,764	19	Regeneration	359	3,500
10	Myocardial infarction	614	6,068	20	Stem cells	357	3,627

**Figure 7:**
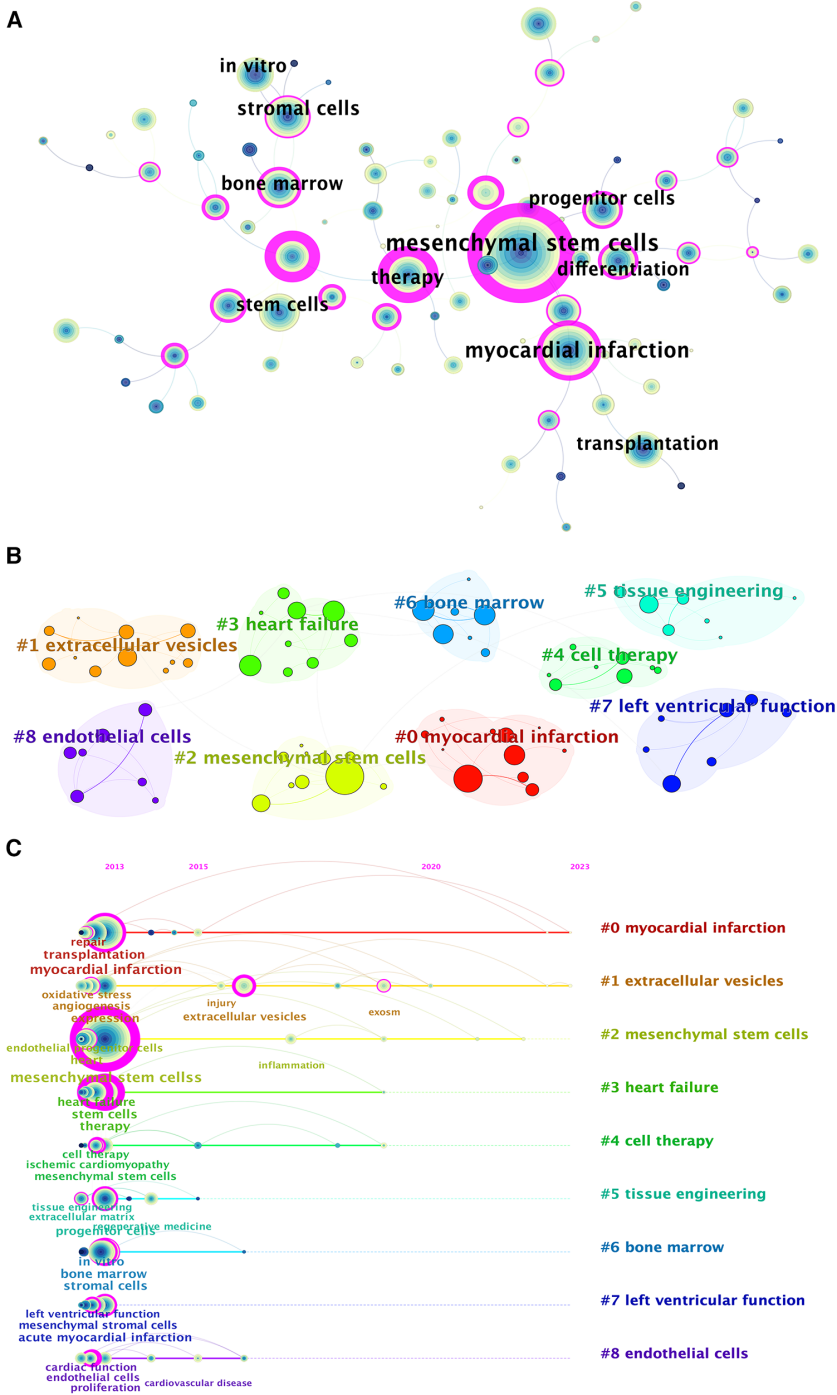
Keyword-related mapping in studies in the field of MSCs in CVDs. (**A**) The figure only shows the top 10 keywords. Map of the keywords in the studies of MSCs in CVDs. (**B**) Subcategories of the main keywords that were utilized in the studies related to MSCs in CVDs. (**C**) A timeline view of keywords based on CiteSpace related to MSCs in CVDs.

Moreover, we applied CiteSpace to group the keywords so that we could illustrate the timeline for keywords after clustering (Figure 7). The association between keywords is manifested in the map (Figure 7A). The larger the node is, the higher the frequency of the keyword was. A point has layers of different colors and thicknesses. The color represents the time of publication. The earlier keywords tend to be bluer; the later ones tend to be yellower. And the thickness represents the number of occurrences during that period.

According to the cluster map (Figure 7B), there are 9 clusters formed: (0) myocardial infarction, (1) EVs, (2) MSCs, (3) heart failure, (4) cell therapy, (5) tissue engineering, (6) bone marrow, (7) left ventricular function, and (8) endothelial cells. Obviously, they are all related to MSCs and exosomes, which to some extent indicate the research center of the intersection of MSCs and cardiovascular fields. By sorting the keywords in the cluster in chronological order based on the year of their first appearance, the timeline view was formed. As is depicted in Figure 7C, the keyword relevant to EVs has maintained a certain number of occurrences from 2013 to 2023, with several outbreaks in a few years. Besides, keywords relevant to myocardial infarction and EVs obtained attention for the longest period on MSCs in CVDs.

**Figure 8:**
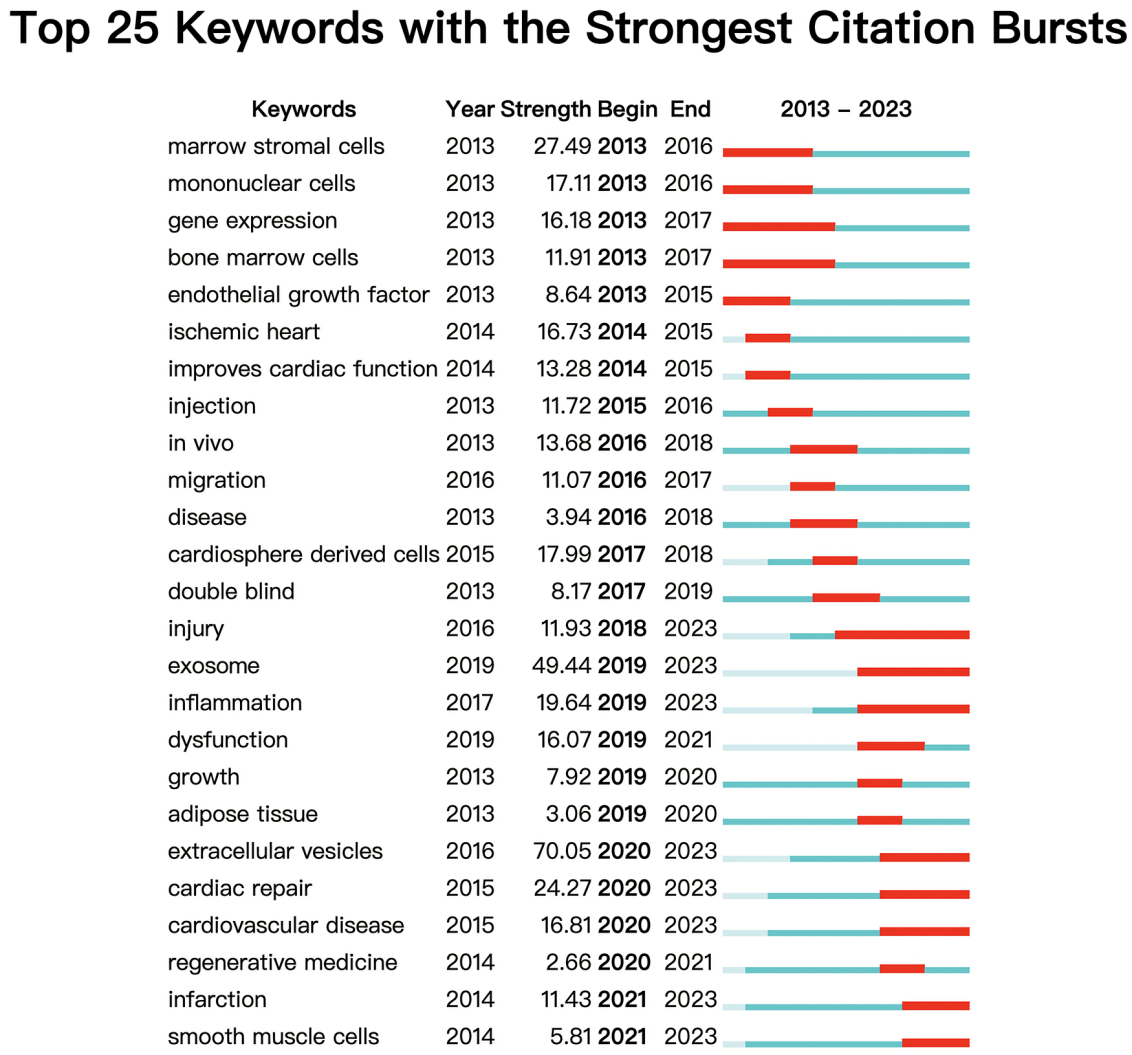
The top 25 keywords related to MSCs in CVDs with the strongest bursts by citeSpace.

Moreover, the top 25 keywords with the strongest citation bursts are depicted in Figure 8. The keyword with the longest burst period was “injury”, which lasted from 2018 to 2023. And the highest burst strength related to MSCs in CVDs was “EVs” (strength = 70.05), “marrow stromal cells” (strength = 27.49), and “cardiac repair” (strength = 24.27). Additionally, “infarction” (2014) and “smooth muscle cells” (2014) have received considerable attention in recent years.

**Figure 9:**
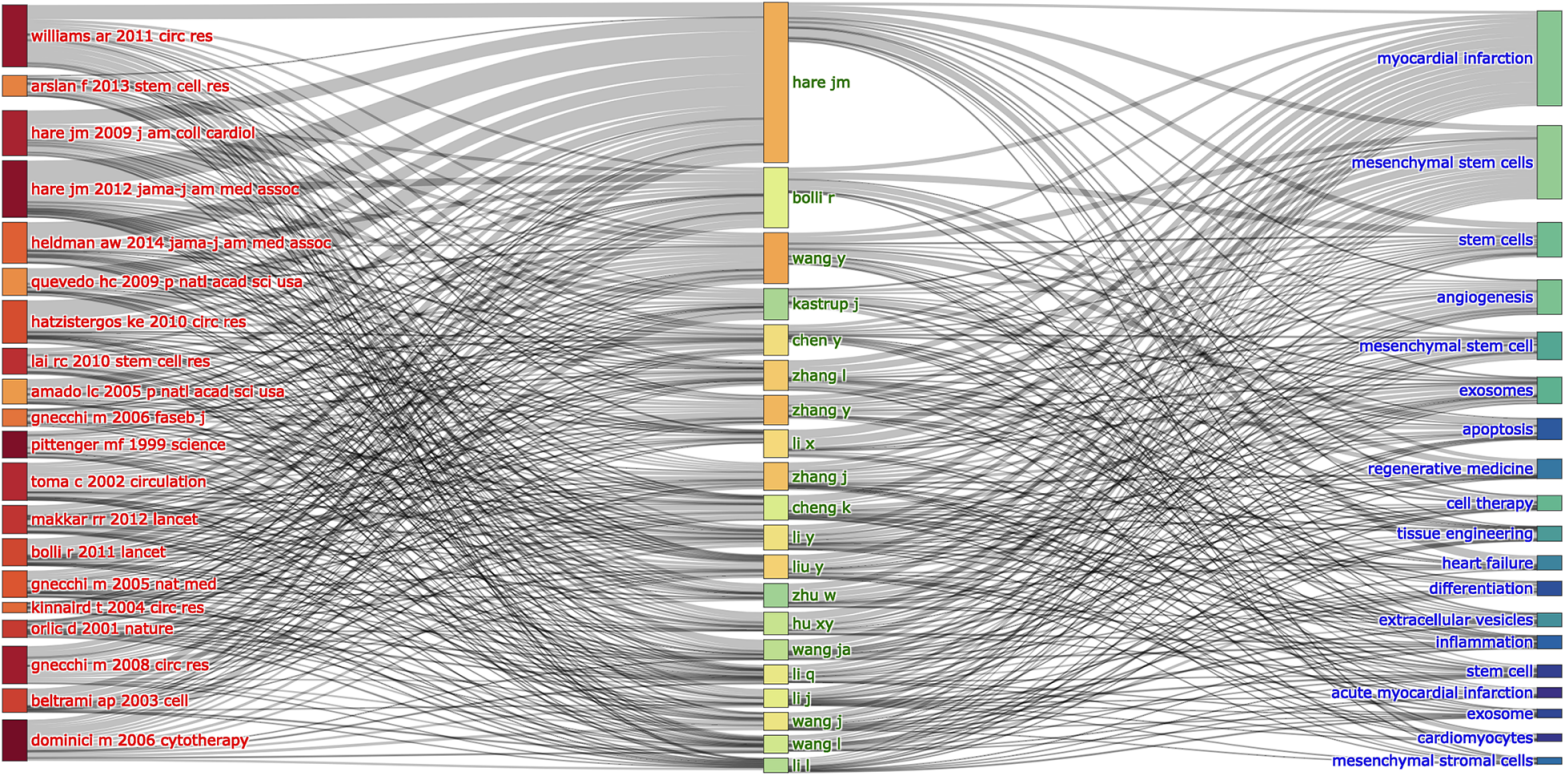
The relationships of the top 20 co-cited references, authors, and keywords evolutions based on an alluvial flow map by R.

The association and connection between the top 20 co-cited references, authors and keywords (only 19 keywords were presented) in MSCs in CVDs are shown in Figure 9 as an alluvial flow map. It depicted that the top 20 co-cited references are more closely related to the research of MSCs, while various types of CVDs occurred (Figure 9).

## 4 Discussion

This study conducts a comprehensive bibliometric analysis utilizing CiteSpace and VOSviewer to investigate recent research developments, identify key research areas, and outline future growth directions in the field of MSCs in CVDs. Through this analysis, we aim to provide a detailed examination of research progress in this domain. By compiling and presenting data on nations and regions, institutions, authors, references, and keywords relevant to this subject, we generate clear and informative tables and figures. These findings are essential for recognizing research hotspots and trends in the field.

### 4.1 Bibliometric information

The founder of the field of MSC biology was Prof. Alexander Friedenstein, who was the first to discover the presence of a group of non-hematopoietic bone marrow stromal cells in the bone marrow, exhibiting clonal adherent growth and morphology similar to fibroblasts (43–45). In 1991, Prof. Arnold Caplan first named these cells as MSCs (46). In 1999, SCIENCE first reported that MSCs have the ability to differentiate into adipocytes, osteoblasts, and chondrocytes simultaneously *in vitro* (41), which ignites everyone’s enthusiasm for studying MSCs, focusing on the various differentiation functions of MSCs. In 2004, Le Blanc et al. reported the successful implementation of the first partially compatible heterogenic MSC transplantation for treating graft-versus-host disease (GVHD) (47). During the period under analysis from 2013 to 2023, this research field garnered significant attention.

In Figure 2, publication and citation reached their peaks in 2018 and 2021, respectively indicating the peak influence of the study during this timeframe. Although subsequent publications maintained a certain quantity, there was a slight decline in research activity levels. The decrease in the number of publications in 2019 may be related to the retraction of 31 papers related to stem cell research in top international journals by Harvard University in 2018. The retraction may have lagged and interfered with the development of stem cell research. However, it is worth noting that the period from 2019 to 2021 coincides with the global pandemic of COVID-19, but the number of publications has still increased to some extent (Figure 2). After analyzing the number of articles and reviews separately, we found that compared to 2019, the number of articles published in 2021 decreased by 2, while the number of reviews increased by 54. This shows that the growth of the number of papers issued during the period of COVID-19 from 2019 to 2021 basically comes from the growth of review type articles. This may be because under the influence of the COVID-19 epidemic, the scientific research work of the laboratory was restricted, but people spent more time writing reviews. This, to a certain extent, also explains the reason for the growth rate of citation numbers is even higher than before the outbreak of COVID-19 in 2019. This also indicates to some extent that the research on MSCs in CVDs has received widespread attention. However, by 2022, the number of articles and reviews were significantly reduced, which may be related to the fact that most countries attach importance to the epidemic and take relatively strict measures to control the COVID-19. And the negative impact of COVID-19 on scientific research may lag behind, and it was concentrated displayed in 2022. The citation count of a specific article dynamically changes over time. We have updated the citation data of the articles we have filtered, and the latest citation data displayed is as of April 23, 2024. As shown in Figure 2, even if only 183 papers in the first eight months were included in the analysis in 2023, the citation count in 2023 has exceeded the citation count of 392 articles in 2022. Therefore, we speculate that the research of stem cells in cardiovascular diseases will continue to develop steadily.

Chinese scholars lead in publication volume, followed by the US, surpassing the third-ranked Italy by nearly sevenfold. Notably, six of the top ten productive institutions are Chinese, with Zhejiang University standing out as the most prolific. As is well known, China has a huge population base, which may be one of the reasons for this result. In addition, China has the highest morbidity and mortality rates of cardiovascular diseases in the world, so it is in urgent need of cardiovascular disease research. While China may not have initiated this research, its subsequent studies and experiences have contributed significantly to the field's advancement.

Furthermore, in Table 4, we can observe that although China has a significant advantage in the number of publications, this advantage becomes less pronounced when evaluating the value of articles based on citation rates. Among the top ten most-cited references, only three were authored by Chinese scholars. Similarly, none of the top ten co-cited references were from Chinese scholars. This indicates that although China is the predominant country in terms of publishing articles in the field of MSCs in CVDs, there are still relatively few high-quality articles published. The possible reason is that Chinese scholars have paid less attention to the mechanism of MSCs, and more attention has been paid to the application and efficacy of MSCs in specific diseases.

Overall, bibliometric analyses indicate a flourishing research landscape on MSCs in CVDs, with countries contributing diverse and in-depth studies. Meanwhile, different countries and laboratories have their own advantages and research focuses, and there are still significant differences in their level in this research field. However, the continuous strengthening of international academic cooperation and exchange is driving rapid progress in this field.

### 4.2 Detection of research hotspots and future direction

#### 4.2.1 Molecular mechanisms of the role by MSCs

Extensive experimental evidence suggests that MSCs play an indispensable role in potentially differentiation into a wide range of cell types, and they likewise have the ability to differentiate into cardiomyocytes or cardiomyocytes-like cells through a variety of strategies that focus on biological, physical, and chemical aspects (48–50). Studies have shown that women with pre-eclampsia will face a higher risk of CVD such as chronic hypertension, ischemic heart disease and late-life stroke (51), and MSC treatment has shown promise in alleviating pre-eclampsia symptoms through immune-modulating, pro-angiogenic, anti-inflammatory and antioxidant effects (52–55). In addition, in the realm of atherosclerosis, BMSCs affect the role of VSMC in plaque formation through different regulatory mechanisms (56, 57). The remarkable differentiation potential of MSCs into various cell types can also promote angiogenesis to enhance tissue perfusion in ischemic conditions (58–63).

The important role of MSCs-derived exosomes in cardiovascular diseases has received widespread attention. Zhang et al. found that the mechanism by which MSC-EXO prevents vascular remodeling in pulmonary hypertension through the regulation of the Wnt5a/BMP signaling pathway (64). In addition, exosomes produced by MSCs can also direct cardiomyocyte differentiation (65–69). The article with most citations (Table 4) has discussed about MSC-derived extracellular vehicles which play an important role in cell-to-cell communication, cell signaling, cell alteration and tissue metabolism at short or long distances in the body (39). Various groups have confirmed that MSC-derived exosomes exhibiting cardio-protective activity are efficacious in animal models of myocardial infarction (70–72), as well as in accelerating wound healing in chronic limb-threatening ischemia (73–76). The most cited literature, Concise Review: MSC-Derived Exosomes for Cell-Free Therapy, also mentioned that MSC-derived exosomes mainly exert their effects through mRNA, miRNA, and proteins, and then alter the activity of target cells through various mechanisms (39).

MiRNA, one of the MSC-sourced bioactive molecules, has the function of regulating gene expression (77–79). As we can see in Figure 8, the keyword “gene expression” has attracted a lot of attention since 2017. Cardiac repair can be enhanced by exosome-derived miRNA gene expression (80–84). By inhibiting the expression of miR-210, Wang et al. demonstrated that miR-210 may inhibit Efna3 at the transcription level, thereby stimulating angiogenesis (85). By comparing fetal dermal MSC EVs with WJ-MSC EVs, Chinnici et al. found that five miRNAs (27b-3p, 125a-5p, 146a-5p, 126-3p and 137) with higher levels have the ability to activate endogenous VEGF-A to promote angiogenesis (86). What's more, Pan et al. incubated LPS-exposed cardiomyocytes with EVs from mouse MSCs, confirming that miR-223-3p in EVs of MSCs can target and inhibit FOXO3 to reduce NLRP3 expression, and subsequently inhibit cardiac inflammation (87). In addition, MSC exo-miRNA has anti-apoptotic function (88–90) and anti-inflammatory function (91), which can enhance the pumping capacity of the heart and effectively treat heart failure (92).

Signaling pathways are essential to cellular physiological activities, and significant proteins can be regulated to modify particular cellular functions (93). In recent years, the role of miRNA in crucial cellular signaling pathways has also been stressed (94–96). Several experiments have demonstrated that miRNAs derived from MSCs regulate multiple signaling pathways, thereby affecting cardiomyocyte repair (97–100). For instance, Sun et al. injected purified exosomes into hypoxia/reoxygenation conditioned rat MIRI model, demonstrating that the MiR-486-5p carried by BMSC-Exos inhibits PTEN expression and activates PI3K/AKT signaling pathway, which ultimately inhibits apoptosis of injured cardiomyocytes (101). In addition, miR-96 in BMSC-Exos promoted BMSC-Exos protection on the cardiac function of DOX-induced rats through inhibiting the Rac1/NF-κB signaling pathway, which reduced free radicals and inhibited the toxicity of DOX, inflammatory reactions and fibrosis (102). The treatment conducted by Zhu et al. treatment with a miR-144 inhibitor in a hypoxic environment could induce increased PTEN expression and decreased p-AKT expression, which led to enhanced apoptosis of H9C2 cells, demonstrating that miR-144 inhibits apoptotic injury under hypoxic conditions by regulating the PTEN/AKT pathway (103). In recent years, a steady stream of pathways associated with miRNA regulation have been identified, but it is still unclear which exosomal miRNAs are the best regulators for the use in the development of MSC-based therapies. By comparing the roles of various types of exosomal miRNAs in the future, it may be possible to find the best therapeutic approaches regarding cardiovascular diseases such as myocardial repair.

MSCs promote healing in ischemic tissue-associated diseases through pro-angiogenic secretion of proteins, again in relation to MSC exosomes, making them a promising resource for regenerative medicine and a wide range of *in vitro* applications (104–106). In addition, a variety of exosome-derived proteases have been shown to favor cardiomyocyte repair and regeneration through multiple mechanisms (107–110). For example, ATP deficiency and initiation of apoptosis during ischemia and reperfusion are supported by proteomic defects in enzymes critical for fatty acid oxidation, glycolysis, and the tricarboxylic acid cycle as well as proteomic excesses of pro-apoptotic proteins. Lai et al. discovered that this glycolytic enzyme deficiency was complemented by an abundance of exosomes from MSCs, and that CD73 could increase survival through activation of the reperfusion injury salvage kinases (111). What's more, MSC-derived exosomes contain multiple proteins involved in signaling molecules, like cytokines, interleukins, chemokines, and growth factors (107). MSCs have been shown to secrete a number of growth factors and cytokines that reduce myocardial ischemia and reperfusion injury (79, 112–116). The therapeutic effects of many cardiovascular diseases are mediated by the reduction of fibrosis, tissue revascularization, cardiomyocyte proliferation, and progenitor cell recruitment, which are required to be mediated by growth factors such as VEGF, HGF, FGF, and neuromodulatory proteins, as well as by cytokines such as colony-stimulating factors and leukemia inhibitory factors (117–121).

In summary, MSCs can not only differentiate directly into cardiomyocytes, but also treat cardiovascular diseases through MSC-derived exosomes. These exosomes can be utilized for their therapeutic potential by leveraging their source miRNAs, proteins, etc., to regulate gene expression, signaling pathways, and growth factors, thus providing a new approach to cardiovascular medicine. By understanding and exploiting the complex interactions facilitated by these exosomes, we can develop more effective and personalized treatments for cardiovascular disease, marking an important step forward in regenerative medicine.

#### 4.2.2 Therapeutic outcome of MSC therapy in CVDs

Recent clinical trials have delved into the efficacy of MSCs in treating CVDs. Clinical trials have demonstrated the safety and efficacy of MSCs in treating CVDs (122–128). MSC therapy has been shown to enhance regional function, tissue perfusion, and reduce fibrotic burden in patients undergoing cardiac surgery (129). Moreover, MSC treatment has proven beneficial for individuals with severe ischemic heart failure and non-ischemic heart disease, leading to improvements in myocardial function, ejection fraction, and quality of life (123, 126–128, 130). In patients with stable heart failure and reduced ejection fraction, MSC therapy has been deemed safe and feasible (124). Additionally, research indicates that MSC therapy may possess antiarrhythmic potential and can improve outcomes in non-ischemic dilated cardiomyopathy patients, irrespective of gender (131, 132). Meanwhile, a SCIENCE trial and a multi-center double-blinded placebo-controlled trial by Qayyum et al. found MSCs to be less effective than expected (133, 134). Correspondingly, the author analyzed that the possible reasons were that the cell retention may have been too short to initiate the paracrine effects, and the treatment may have been initiated too late. These findings underscore the promise of MSC therapy in managing a range of heart conditions and indicate the need for further exploration in larger clinical trials.

#### 4.2.3 Trend of MSC application in CVDs

**Figure 10:**
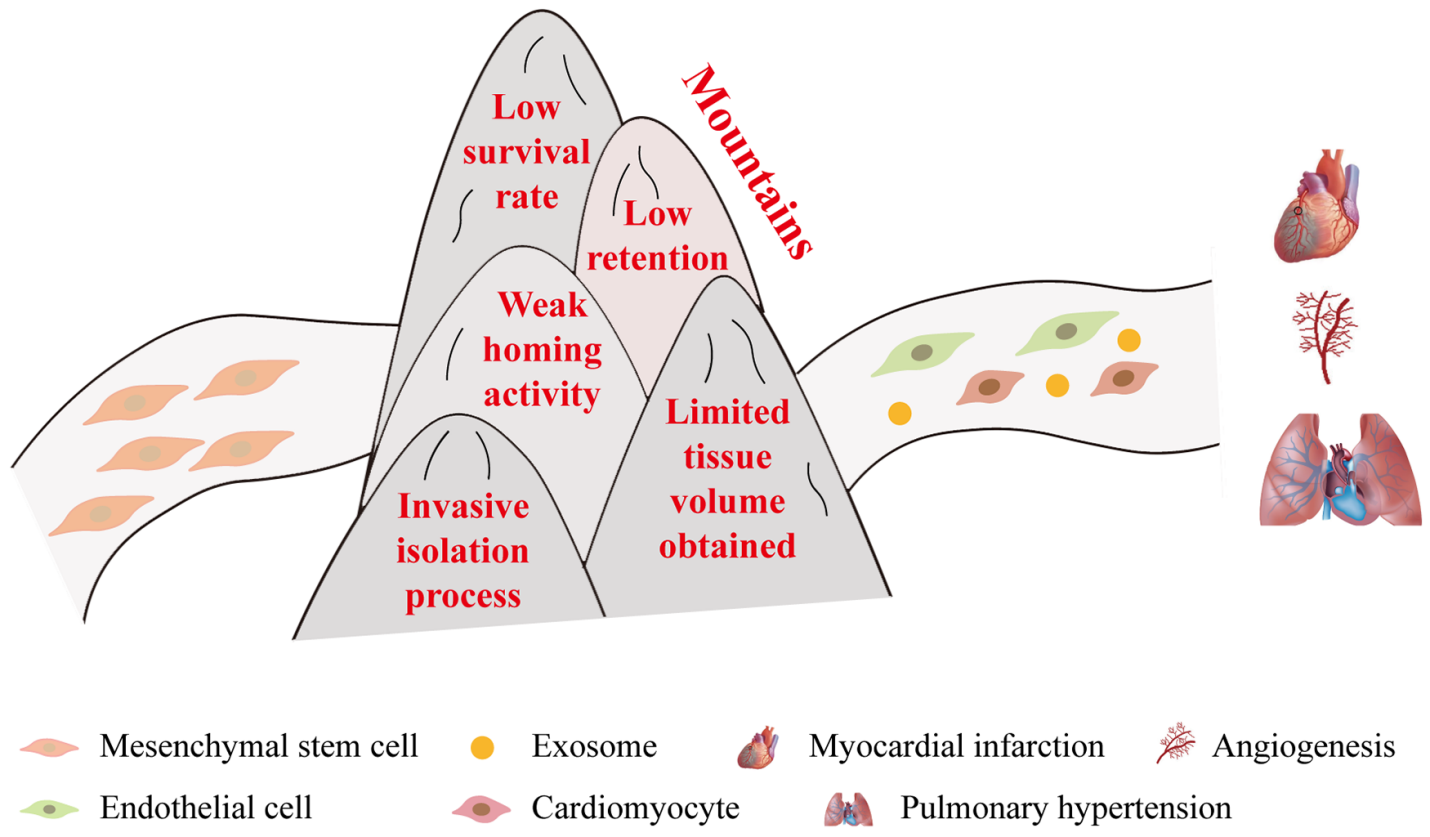
Challenges faced by MSCs in clinical applications. In order to realize the treatment of diseases like myocardial infarction and pulmonary hypertension, MSCs need to overcome the following five significant obstacles: (1) low survival rate, (2) low retention, (3) weak homing activity, (4) invasive isolation process, and (5) limited tissue volume obtained.

MSCs, with their broad origin, robust differentiation capacity, and regenerative potential, play an important role in regenerative medicine (135, 136). They have long been a focal point in clinical research for treating CVDs (137). The therapeutic effects of MSCs on various CVDs, including myocardial infarction, atherosclerosis, pulmonary hypertension, and so on, have been extensively investigated (22, 56, 138–141). The immense potential of MSCs in various applications remains undeniable in the future. However, recent research on MSCs may have reached a plateau, and the problems of low survival after transplantation and low differentiation efficiency still need to be solved (138, 142). What’s more, low retention, weak homing activity, invasive isolation process, and limited tissue volume obtained are also the key issues that limit the application of MSCs in the clinic (Figure 10) (143–145).

Indeed, compared to MSC cell therapy, cell-free treatments based on MSC secretomes and EVs in show significant potential in regenerative medicine (146–150). EVs have proven to be an effective drug delivery method for a variety of conditions, including tumors, neurodegenerative disorders, renal disease, and CVDs (151–157). Additionally, biological effectors can be transferred by EVs through natural or engineered means. MSC-derived EV-based therapy provides several benefits over MSC-based cell therapy, according to a growing number of studies: low immunogenicity, low tumorigenicity, low cytotoxicity, easy preservation, high stability, low degradability, blood–brain barrier penetration ability, possible intravenous injection, internalization into cells, potential cargo loading ability, intrinsic homing properties, immunotolerance properties, absence of genotoxic effects, and no endotoxin production (158, 159). Laboratory studies have shown that compared to MSCs, MSC-derived exosomes and their cargos also play an important role in cardiovascular diseases, which has been introduced in Section 4.2.1. So far, several clinical studies have evaluated the efficacy of EVs derived from MSCs in respiratory diseases (160–164). However, the efficacy of MSC-derived EVs in CVDs should be further explored.

Currently, biomaterials technology combined with cellular therapy plays a huge role in cardiovascular diseases (165, 166). For example, treatments with collagen can promote cardiomyogenic differentiation of MSCs (167, 168). In recent years, numerous nanomaterials have also been gradually combined with MSC therapy (169, 170). Studies have found that graphene as a culture substrate contributes to the survival rate of MSCs after transplantation (171, 172). In addition, hydrogels can be used as scaffolds to induce MSC differentiation (173, 174). Embedding MSCs in hydrogels for treatment can promote wound healing and angiogenesis, as well as anti-inflammatory effects, which are crucial in the treatment of cardiovascular diseases (175–178). In addition, in terms of non-extracellular matrix based, the use of cellular derivatives of MSCs as polymeric biomaterials (e.g., collagen, gelatin, hyaluronic acid, chitosan, etc.) for scaffold fabrication for cardiomyocytes can influence stem cell behavior (179–182).

### 4.3 Limitations

In this study, we collected data from WoSCC through a retrieval strategy and analyzed it, thus obtaining certain reliable results. However, it is essential to acknowledge certain limitations. Firstly, the retrieval algorithm that served as the method to get our results may have ineluctable flaws, potentially leading to omissions in retrieving relevant literature. Secondly, relying solely on data from a single database restricted the scope of our study results. What's more, due to time constraints in obtaining retrieval literature, it was inevitable that some recent articles were not included in our research.

To explore the latest trends and key areas of interest concerning MSCs in CVDs, future research should encompass corresponding bibliometric analyses. Nonetheless, this bibliometric study conducted a meticulous analysis and thorough examination of the literature data, providing valuable insights into the progression of MSCs in CVDs. It also underscored promising directions for future development and research hotspots within the field.

## 5 Conclusion

Over recent years, research on MSCs has emerged as a beacon of hope across a wide spectrum of diseases, notably in the realm of CVDs. Our study, as a bibliometric analysis, has synthesized the latest advancements, identified the research hotspots, and deliberated on the prospects in the field of MSCs in CVDs. Currently, investigating the molecular mechanism underlying the role of MSCs in CVDs stands out as a prominent research trend. Noteworthy topics include the study of MSC-derived exosomes, regulation of gene expression and signaling pathways by miRNAs, and promotion of growth factors are also hot topics. In addition, the utilization of biomaterials plays a critical role in facilitating the transplantation and functionality of MSCs. By holding an escalating trend in international and inter-institutional collaborations, this field of research is poised to remain a key area of study, with a primary focus on advancing treatments for CVDs and enhancing longevity.

## Data Availability

The original contributions presented in the study are included in the article/Supplementary Material, further inquiries can be directed to the corresponding authors.
